# Evaluation of Implementation Level of Community Health Strategy and Its Influence on Uptake of Skilled Delivery in Lurambi Sub County-Kenya

**DOI:** 10.24248/eahrj.v4i1.623

**Published:** 2020-06-26

**Authors:** Rose Muhanda, Vincent Were, Henry Oyugi, Dan Kaseje

**Affiliations:** a Department of Community Health and Development, Tropical Institute of Community Development, Great Lakes University of Kisumu, Kisumu, Kenya; b Kenya Medical Research Institute, Centre for Global Health Research, Kisumu, Kenya

## Abstract

**Background:**

Despite the widespread application of the community health strategy (CHS) in Kenya and evidence of its effectiveness in reducing health outcomes at the household level, data from Kakamega County, of which Lurambi sub-county is part of, still showed that skilled birth delivery was at 47% against the national estimateof 62% and a target of 90%. However, there was limited evidence on the level of CHS implementation and its association with the uptake of skilled delivery.

**Methods:**

The study employed a cross-sectional analytic design. A structured validated community unit (CU) scorecard and a household questionnaire were used to collect quantitative data from the CUs through Community Health Extension Workers (CHEWs) and at the household level through mothers with children below 1 year. A random sample of 436 mothers from all the 38 Community Units (CUs) was included. CU functionality was assessed using 17 binary indicators (scored as 1 for a positive response, 0 otherwise) and total scores were expressed as percentages. Fully functional CUs scored ≥80% and semi-functional CU scored >50 to <80%. No CU was non-functional (scored ≤50%). Data from the CUs were merged with data at the household level. Association between CU functionality and skilled delivery was assessed using multivariable binary logistic regression controlling for socio-demographic variables. Adjusted Odds Ratios (OR) and 95% Confidence Intervals (95%CI) are reported.

**Results:**

A total of 38 CUs were assessed and of these, 26(68.6%) were fully functional and 12(31.4%) were semi-functional, 18(47.4%) had both household registers (MOH 513) and service delivery logbooks (MOH 514). Overall, 387(80.0%) of mothers had skilled birth deliveries, 263(68%) were from functional CUs and 124(32%) were from semi-functional CUs. Pregnant women were more likely to have skilled deliveries in fully functional CUs than semi-functional CUs (OR=1.3; 95% CI=1.1-2.4; p-value<.001). Other factors significantly associated with uptake of skilled delivery included receiving health education(OR=2.9;95%CI =1.4-6.1, p=.005), being visited at least twice by Community Health Volunteers, CHVs(OR=1.9;95%CI=1.1-3.5, p=.045), attending antenatal care clinics, ANC (OR=3.4;95%CI=1.3-3.5, p=.012), receiving advice where to deliver (OR=4.1;95%CI=1.8-9.4, p=.001).

**Conclusion:**

2 out of 3 community units were fully functional, and functionality was associated with increased uptake of skilled delivery. In a fully functional CUs, Community Health Volunteers provided health education through regular visits and they were able to provide a referral to health facilities for the pregnant women. To achieve national targets for skilled deliveries and universal health coverage, there is a need to ensure CUs are fully functional

## BACKGROUND

Globally, one-third (34%) of births take place at home without the help of a professional skilled birth attendant^1^ which means that 45 million births are occurring at home without skilled health personnel each year.^2^ A range of healthcare services throughout pregnancy, childbirth and the postnatal period is key to improving maternal and newborn health to reduce maternal and child morbidity and mortality.^3^ Skilled attendants assist in more than 99% of births in developed countries compared with 62% in developing countries.^1^ Presence of skilled medical personnel at child delivery is one of the key indicators towards the Millennium Development Goals (MDGs)5 of improving maternal health and sustainable development globally to reduce maternalmortality ratio to less than 70 per 100,000 live births.^4-6^ Globally, the goal is to have 80% of all births assisted by skilled medical attendants by 2005, 85% by 2010 and 90% by 2015.^5^. A study from South India had shown that skilled assistance during delivery can reduce the risk of obstructed labour and it is highly associated with the place of delivery.^7^ In Africa, reports show that less than 50% of births are attended by a skilled health worker.^6,8^ In Kenya, coverage of skilled deliveries was 62% while 61% of births were delivered at a health facility by 2014 while the maternal mortality ratio was 362 per 100,000 live births.^6^ Neonatal deaths contributed to 60% of Kenya's infant mortality rate (52/1000 live births).^9-14^

Prevention of maternal deaths and neonatal deaths can be achieved by ensuring that mothers deliver in the presence of skilled medical personnel. In Kenya's National Health Sector Strategic Plan (NHSSP II–2005–2010), a new approach to the delivery of health care services, known as the Kenya Essential Package for Health (KEPH) was outlined. The KEPH has six life-cycle cohorts and six service delivery levels.^14^ One of the key innovations of KEPH was the introduction of level 1 service, which was aimed at empowering Kenyan households and communities to take charge of improving their own health.^14^ In line with the vision 2030, the government intended to scale up community units in the country, and also work towards improving the health service delivery at level one, (household level).^14^ In 2007, the Kenya Ministry of Public Health and Sanitation adopted a community health strategy aimed at reversing the poor health outcomes to meet the Millennium Development Goals 4 and 5^15^.

The strategy also aimed at strengthening community participation and encouraging communities to take action towards their health through functional community units and linked health facilities. Community Health Strategy (CHS) is an approach to health care service delivery in Kenya by the Health Sector as an effort to revitalise Comprehensive Primary Health Care and also aimed to improves access to health care and health service indicators such as skilled delivery.^14^

The process of establishment of a community unit has been described adequately else where.^10,16-18^ In brief, it involves; The formation of committees at the community and health facility levels as governance/linkage structures; The identification and training of community health workers to support households in health improvement initiatives, as well as to maintain a village register, and facilitate dialogue at the household level; identification, training and deployment of community health extension workers (CHEWs) for each community unit as the facilitator of dialogue at the community level and supporter of CHVs. The information collected in the household registers is to be updated every six months by the community health volunteers. CHVs and CHEWs also dialogue sessions with community members and at households. The dialogue sessions are facilitated by CHVs during home visits and by CHEWs at general community meetings, while the health facility staff are expected to facilitate dialogue at the management committee meetings and the Sub-county Health Management Teams (SCHMTs) facilitated at sub-county health stakeholder forums.^10,16-18^

Ager et al developed a Community Health Unit (CHU)functionality scorecard and categorised CHUs as functional, Semi-functional, or Non-Functional.^16^ A set of 17 indicators are used in the scoring and percentage score are generated. A positive score is assigned 1 and a negative score is assigned 0. The scores are then summed up and expressed as a percentage out of 17 indicators per CHU. Functional CHUs are those that score at least 80%, semi-functional CHUs scores ≥50% and <80% and non-functional CHU scores ≤50%.

However, despite the widespread application of the community health strategy in Kenya since its inception in 2006 and evidence that Community Health Strategy is effective in reducing health outcomes at household level especially where it is fully functional, skilled delivery in Kakamega County is still low at 47% compared to a national estimate of 62 %^4^ which was still below the target of 90% of deliveries by 2015 and also still below the targets of Sustainable Development Goals of 90% skilled deliveries by 2030.^4^ A study on the effectiveness of the community health strategy on health outcomes in Butere district established that when components of the strategy were fully implemented and sustained in different socio-demographic contexts, then a participatory community planning based on household information drives improvement of health indicators.^18^ There is limited data on levels of functionality of community units and at its influence on the uptake of skilled birth deliveries in Lurambi Sub-County. In this study, we hypothesized that mothers were more likely to have skilled deliveries if they were residing in a fully functional community unit than if they were residents of non-functional or semi-functional community units.

## METHODS

### Study Site

The study was undertaken in Lurambi Sub County in Kakamega County of Western Kenya. Lurambi sub-county has an estimated population of approximately 160,229 living in the area of about 161.8 square Kilometres. The sub-county just like the rest of the county depends primarily on agriculture and most farmers grow sugarcane as the main cash crop. Most of the food crops are grown on a small scale annually. The main crops are sugarcane, maize, bean, cassava, finger millet, and sorghum. Maize forms the staple food for the sub-county. It has 53 health facilities out of which 20 are public, 29 are private and 4 are faith-based or owned by non-governmental organisations

### Study Design

This was an analytic cross-sectional study using quantitative methods of data collection and had two components. The first component assessed the functionality of Community Health Units and the second component assessed the association of CHU functionality and skilled delivery. The study was conducted both at the community unit and at household levels.

### Study Population

The study focused on all 38 Community Health Units within the Lurambi sub-county. The Community Health Extension Workers (CHEWs) were the respondents for the CHU assessment. A total of 38 CHEWs were interviewed, one in each CHU. The Community Health Units were assessed based on their functionality concerning 5 parameters: Training, Community Health committees, Community Based Health Information System (CBHIS), Community Dialogue and Community Action Days. The household survey focused on the women with children below 1 year (n=6871)

### Sample Size and Sampling Technique

Lurambi sub-county had 38 Community Health Units and all were included in the assessment hence total coverage. The sample size for the mothers was calculated using an estimated skilled delivery prevalence of 47% for Kakamega County for Z-score of 1.96 at a 95% Confidence Interval and a 5% Level of Precision. The minimum sample size was 382 and a maximum target of 458 having added a non-response rate of 20%. A Stratified sampling technique was used to sample women from functional and semi-functional Community Health Units. The total number of Functional Community Units was first determined, and villages were selected from each of the community units using a simple random sampling method. A list of women with children below 1 year was obtained from the household register from each village within the community units. The list was organised per village and community unit. Women who delivered less than 1 year, or who are not usual residents of the study areaor those who delivered outside the study area were excluded.

All the 38 Community Health Units in the Lurambi sub-county were included. A simple random sampling technique was used to select eligible respondents from the list of women with children below 1 year in the sampled villages. At least 30% of the villages were sampled.

### Data Collection

The study used structured questionnaires for household inter views and a checklist for functionality assessment as the main data collection instruments. The research assistants were recruited, selected and trained on study procedures which included questionnaire content and on ethical considerations. The training took two days. The household interviews were conducted at the household levels amongst the eligible mothers. Interviews were conducted in Kiswahili for all respondents. The assessment assessing the functionality of Community Units was done using the CHUs functionality scorecard. This tool was administered at the linked health facilities where key informal interviews were conducted with health facility personnel in charge. A tool for assessing the functionality of community health units was adopted from the African Medical and Research Foundation.^16^ The tool has 5 parameters which include Training of Community health volunteers for 10 days, Community Health Committee for 5days training, CHC meetings, holding community dialogue monthly and action days and lastly ensuring that the Community-Based Health Information System is always updated. These 5 parameters are further broken down into 17 elements (Table 4) which are scored 1 for a positive response and 0 for a negative response. The scores are summed up and percentages calculated based on the number of positive responses out of 17 elements. A functionality categorisation is them used to classify whether the CUs are functional, semi-function or non-functional. If the overall percentage score is ≥80% then CU is Fully function, between>50% to <80% is semi-function and ≤50% is non-functional.^16^

**TABLE 1.** Distribution of the Study Population by Socio-Demographic Factors and Functionality of Community Units

Demographic information	Overall (N=436) n (%)	Functional (n=299) n (%)	Semi-functional (n=137) n (%)	Fisher Exact test P-Value
**Age in years**				**0.304**
16 – 24	156(35.8)	104(34.8)	52(38.0)	
25 – 35	217(49.8)	150 (50.2)	67(48.9)	
36 – 44	60 (13.8)	43(14.3)	17(12.4)	
45 – 55	3(0.7)	2(0.7)	1(0.7)	
**Level of Education**				**0.481**
Primary	181(41.5)	118(39.5)	63(46.0)	
Secondary	171(39.2)	114(38.1)	57(41.6)	
Tertiary	74(17.0)	59(19.7)	15(10.9)	
None	10(2.3)	8(2.7)	2(1.5)	
**Time to health Centre**				**0.107**
Less than 1 hour	336(77.1)	228(76.3)	108(78.8)	
1-5 hours	95(21.8)	67(22.4)	28(20.4)	
Over 5 hours	5(1.1)	4(1.3)	1(0.7)	
**Occupation**				**0.374**
Farmer	88(21.9)	57(20.4)	31(25.4)	
Daily labourer	49(12.2)	35(12.5)	14(11.5)	
Self-employed	102(25.4)	76(27.3)	26(21.3)	
Housewife	129(32.2)	84(30.1)	45(36.9)	
Civil servant	33(8.2)	27(9.7)	6(4.9)	
**Occupation of husband**				**0.167**
Farmer	102(23.4)	63(21.0)	39(28.4)	
Daily labourer	144(33.0)	98(32.8)	46(33.6)	
Business	132(30.3)	90(30.1)	42(30.7)	
Civil servant	58(13.3)	48(16.1)	10(7.3)	
**Husband's education level**				**0.207**
Primary	155(35.6)	101(33.8)	54(39.4)	
Secondary	179(41.0)	116(38.8)	63(46.0)	
Tertiary	99(22.7)	79(26.4)	20(14.6)	
None	3(0.7)	3(1.0)	0(00.0)	

Descriptive statistics on the association of community health strategy and functionality

**TABLE 2.** Classification of community health strategy elements by level of community unit functionality

Community Health Strategy elements	Overall (N=38) n (%)	Functional n (%)	Semi-functional n (%)	Fisher's Exact Test P-Value
**Two Trained CHEWs**				**0.065**
Yes	34(89.5)	26(96.3)	8(72.7)	
No	4(10.5)	1(3.7)	3(27.3)	
**Existence of trained CHC (1-13)**				**0.065**
Yes	34(89.5)	26(96.3)	8(72.7)	
No	4(10.5)	1(3.7)	3(27.3)	
**Trained CHVs as per the Population**				**0.074**
Yes	34(89.5)	24(88.9)	10(90.9)	
No	4(10.5)	3(11.1)	1(9.1)	
**CHVs have MOH 513aand 514^b^**				**0.110**
Yes	18(47.4)	15(55.6)	3(27.3)	
No	20(52.6)	12(44.4)	8(72.7)	
**Availability of MOH 516^c^**				**0.326**
Yes	34(89.5)	25(92.6)	9(81.8)	
No	4(10.5)	2(7.4)	2(18.2)	
**All CHVs have referral booklets MOH 100^d^**				**0.024**
Yes	25(65.8)	21(77.8)	4(36.4)	
No	13(34.2)	6(22.2)	7(63.6)	
**Quarterly CHC meetings are held**				**0.0653**
Yes	35(92.1)	25(92.6)	10(90.9)	
No	3(7.9)	2(7.4)	1(9.1)	
**CHVs hold monthly meetings**				**NA**
Yes	38(100.0)	27(100.0)	11(100.0)	
No	0(00.0)	0(00.0)	0(00.0)	
**Monthly dialogue days are held**				**0.289**
Yes	37(97.4)	27(100.0)	10(90.9)	
No	1(2.6)	0(0.0)	1(9.1)	
**Quarterly action days are held**				**0.078**
Yes	37(97.4)	27(100.0)	10(90.9)	
No	1(2.6)	0(0.0)	1(9.1)	

a MOH 513: Household Register

b MOH 514: Service delivery logbook.

c MOH 516: Community Chalk Board

d MOH 100: Referral booklet

**TABLE 3.** The Influence of Community Health Strategy Elements on Uptake of Skilled Delivery

Community Health Strategy	Overall (N=436) n (%)	Skilled Delivery n (%)	Unskilled Delivery n(%)	Adjusted Odds Ratio (95% CI)	P values
**Functionality**					
Functional	299(68.6)	263(68.0)	36(32)	3.0(1.8-4.9)	<.001*
Semi-functional	137(31.4)	124(32.0)	13(68)	Ref	
**Received Health Education**					
Yes	403(92.4)	366(94.6)	37(5.4)	2.9(1.4-6.1)	.005*
No	33(7.6)	21(5.4)	12(94.6)	Ref	
**Times Visited by CHVs**					
None	74(17.0)	62(16.1)	12(83.9)	Ref	
Twice	142(32.6)	129(33.3)	13(66.7)	1.3(0.7-2.5)	.394
More than twice	220(50.4)	196(50.6)	24(49.4)	1.9(1.1-3.5)	.045*
**Attended ANC**					
Yes	418(95.9)	376(97.2)	42(2.8)	3.4(1.3-9.0)	.012*
No	18(4.1)	11(2.8)	7(97.2)	Ref	
**Received Advice Where To Deliver**					
Yes	411(94.3)	376(97.2)	35(2.8)	4.1(1.8-9.4)	.001*
No	25(5.7)	11(2.8)	14(97.2)	Ref	
**Having Health Insurance Cover**					
Yes	101(23.2)	99(25.6)	2(74.4)	2.4(1.2-4.7)	.011*
No	335(76.8)	288(74.4)	47(25.6)	Ref	
**CHV Referred You To Hospital for Delivery**					
Yes	253(58.0)	236(61.0)	17(39)	1.8(1.1-3.0)	.012*
No	183(42.0)	151(39.0)	32(61)	Ref	
**Who Decided Where You Were To Give Birth**					
Myself	186(42.7)	160(41.4)	26(58.6)	Ref	
My husband	45(10.3)	34(8.8)	11(91.2)	0.4(0.1-1.0)	.059
Self and husband	162(37.2)	153(39.5)	9(60.5)	0.3(0.1-0.9)	.026*
CHV	43(9.9)	40(10.3)	3(89.7)	0.5(0.2-1.5)	.208
**Difference Between Giving Birth at Health Facility**					
Yes	407(93.3)	370(95.6)	37(4.4)	6.1(2.2-16.9)	.001*
No	13(3.0)	9(2.3)	4(97.7)	1.1(0.3-4.8)	.897
Don't know	16(3.7)	8(2.1)	8(97.9)	Ref	

The multivariable logistics regression was used to generate adjusted odds ratios. The variables adjusted for are in Table 1 considered confounder for the uptake of skilled deliveries

**TABLE 4.** The 17 Functionality Elements Of A Community Health Unit Organised Sequentially To Represent The Journey That It Follows From Inception To Maturity

no	Components	Yes(1), No(0)
1	CHEWs trained	
2	CHC trained	
3	CHVs trained	
4	CHVs supplied with CHV kits	
5	All trained CHVs have MoH 514	
6	CHV reporting rate above 80%	
7	CHU has a chalkboard	
8	All trained CHVs have referral booklets	
9	CHU action plan developed	
10	Quarterly CHC Meeting held	
11	CHVs monthly Meetings	
12	All reporting CHVs (MoH 514) receiving stipend	
13	Monthly dialogue days held	
14	Quarterly Health Action Days held	
15	DHMT supervisory visit conducted	
16	CHU has bicycles for use by CHVs	
17	CHU having a sustainable initiative(IGAs)	
	Total Score out of 17	xx
	Percentage (%) Score	xx

Key	Functionality	Categorisation
Yes-Fulfilled/positive (Score one¬1)	≥80%	Functional
No-Not fulfilled/negative (Score zero¬0)	>50 to <80%	Semi-Functional
	≤50%	Non-Functional

Note: The three (3) cardinal elements (15, 16, 17) MUST all be fulfilled for a CU with ≥80% score to be functional

The data collection period of reference was October 2015 to September 2016. Focal persons were interviewed on the checklist for functionality. These are contact persons of the CHUs. For the household interviews, a list of eligible mothers was obtained from CHVs based on the household registers from different villages and those who consented were interviewed

### Data Analysis

The functionality of the CUs was done using a score sheet from a checklist where the scores were classified as fully functional, semi-functional or not functional. All mothers were linked to the specific CUs through the village of residence. Descriptive statistics wereused to assess functionality. Functionality (full versus semi) of the CU was the main independent variable while skilled delivery was the main outcome. There was no non-functional CUs as per the classification. Chi-square test and logistic regression were used to assess the association between functionality and skilled birth deliveries. Odds ratio at 95 % Confidence Intervals (OR, 95%CI) was reported while p-value<.05 denoted significant results. Data entry and analysis were done using statistical package for social sciences version 20(IBM Corp. Released 2011. IBM SPSS Statistics for Windows, Version 20.0. Armonk, NY: IBM Corp).

### Ethical Considerations

Approval to carry out the study was obtained from Great Lakes University of Kisumu Ethical Committee (Ref: No. GREC/018/256/2016), Permission tp carry out the study was also approved by the National Commission for Science, Technology, and Innovation (ref: NACOSTI/p/17/74545/17004), Kakamega County Health Management Team and National Commission for Science and Technology. Permission to conduct this study was also received from the Lurambi Sub County Health Management Team and the local administration to conduct interviews. Written informed consent was obtained from respondents and each one of them was informed of their rights and obligations. They were informed that there was no risk; legal, social or psychological of participating in the study and participation was purely voluntary. The data collected from study participants were kept strictly confidential in password-protected computers only accessible to the investigators. Participants were only identified by a code. No names of participants were collected in this study.

## RESULTS

### Socio-demographic characteristic of participants and Community Units

A total of 436 women were interviewed. Of this 156(35.8%) were aged between 16 and 24,217(49.8%) were aged between 25 and 35 years while the minority 3(0.7%) were aged between 45 and 55 years. On the level of education, 181(41.5%) had only primary education, 181(39.2%) had secondary education, 74(17.0%) had tertiary education and 10(2.3%) had no education. Over three-quarters of the respondents, 336(77.1%) took less than an hour to get to a nearby health facility, 95 (21.8%) took between 1 and 5 hours and 15(.1%) took over 5 hours. One third, 129(32.2%) of the respondents were housewives, 88(21.9%) were farmers, 49(12.2%) were daily labourers, 102(25.4%) were self-employed and 33(8.2%) were civil servants. On respondent's husband's level of education, 155(35.6%) had only primary education, 41.0% had secondary education, 99(22.7%) and minority 3(0.7%) had tertiary education and no education respectively (Table 1).

### Classification of Community Health Strategy Elements by Level of Community Unit Functionality

Of the 38 CUs assessed, 26(68.4%) were fully functional while 12(31.6%) was semi-functional. No community unit was not functional. Out of the key elements of Community health strategy, the Community Health Volunteers held all regular monthly meetings 38(100%) as required. Dialogue and action days were also held in both functional and semi-functional units. However, training of CHEWs, the existence of trained Community Health Committees, and trained Community Health Volunteers all stood at 34(89.5%). There was a shortage of reporting tools (MOH 513 and MOH 514) in both functional and semi-functional units at 15(55.6%) and 3(27.3%) respectively. On referral booklets (MOH100), 13(34.2%) of all CHVs did not have booklets (Table 2)

### The Influence of Community Health Strategy Elements on Uptake of Skilled Delivery

Pregnant women from functional community health units 296(68%) were more likely than those who were from semi-functional 146(32%) to have skilled delivery (OR=3.0; 95% CI, 1.8-4.9, p<.001). About 366(94.6%) of those who had received health education were more likely to uptake skilled delivery as compared to those who did not receive health education (OR=2.9; 95% CI, 1.4-6.1, p=.005). Pregnant women who were visited by CHV's more than twice (50.6 %) were more likely to have skilled delivery than those who were not visited 196(50.6%)(OR=1.9; 95%CI, 1.0-3.5, p=.045). Pregnant women who attended ANC, 76(97.2%) (OR=3.4; 95% CI, 1.3-9.0, p=.012) and those who received advice on where to deliver (376 (97.2%)(OR=4.1; 95% CI, 1.8-9.4; p=.001) were more likely to have skilled delivery as compared to those who did not attend ANC or those who did not receive advice on where to deliver respectively (Table 3)

## DISCUSSION

### Implementation Levels of Community Health Strategy

The study established that most of the community health units (68.6%) in Lurambi Sub County meet at least 80% functionality assessment scores of the strategy and are fully functional while the rest of the CUs are semi-functional. This shows higher levels of implementation compared to report on community health services in Kenya conducted in Turkana, Kibera, and Machakos which established that only 28% were fully functional and the majority were semi-functional.^15^ The study has also established that mothers living within fully functional CUs were more likely to have skilled delivery than those from semi-functional CUs. This finding is supported by findings of AMREF Health Africa in one of its programs in Makueni County which was using the CHUs and they reported that skilled birth delivery improved from 37.5% to 44.2 % in 12 months.^16^ The Community Strategy had set an ambitious target of reaching 16 million Kenyans (3.2 million households) being enrolled by 2009 which was yet to be achieved by 2017. The strategy also aimed at strengthening community participation and encouraging communities to take action towards their health through functional community units and linked health facilities. Community Health Strategy (CHS) is an approach to health care service delivery in Kenya by the Health Sector as an effort to revitalize Comprehensive Primary Health Care. Community Health Strategy performs a key role in the renewal of comprehensive Primary Health Care in Kenya. It improves access to health care thus improves health service indicators such as skilled delivery.^14^ The community health strategy introduced the community-based approach as the mechanism through which households and communities take an active role in health and health-related development issues. The primary approach was to establish Community Health Units (CUs) to serve a local population of 5,000 people, enlisting Community Health Volunteers (CHVs) who each are directly responsible for the delivery of services to the communities.^19^

According to the Kenya vision 2030 and the second National Health sector strategic plan, the community health strategy approach ensures that Kenyan communities have the capacity and motivation to take up the essential role in health care delivery.^16^ This study established that the program has made progress in implementation level by addressing issues pertinent to the community health strategy such as; Having 2 trained CHEWs, the existence of trained CHC, trained CHVs, availability of MOH 516, MOH 513, MOH 514 and MOH 100, SCHMT supervision, Community Health Volunteers monthly meetings, Community Health Volunteer reporting rate of above 80%, quarterly CHC meetings, quarterly dialogue days and quarterly action days.

### Level of Uptake of Skilled Delivery

This study has established that 80% of the skilled deliveries were in the health facilities and 68.6% of women who had skilled delivery were more likely to be from fully functional units than those from semi-functional. The results also revealed that 92.4% of the women received health education, which might have helped in knowing the risks and benefits of skilled delivery. This finding is supported by a study in Ghana which established that there is a statistically significant association between women's health education and skilled delivery.^20^

The high prevalence of skilled deliveries could be attributed to the messages and influence of community health volunteers.

The study further established that 94.6% of the women who received health education on skilled delivery were more likely to have skilled delivery compared to those who did not receive health education. This is because those who are educated are aware of the risks of giving birth in an unskilled manner. A study also found that mothers who are educated can make wise decisions about their health than their counterparts; this is consistent with the findings of this study.^21^ The results showed that women were 4.2 times more likely to deliver in hospitals from Community Units with 2 CHVs trained than those who are not.

A report on Community Health in Kibera Kenya also emphasised that women who were frequently visited by CHVs are more likely to have skilled delivery since CHVs encourage women to go to health facilities during delivery.^22^ This finding is was also consistent with the findings where CHWs had a great impact on increasing the uptake of health services.^22^

In a multivariate logistic analysis of the variables, it was established that a fully functional community unit, trained Community Health Volunteers, trained Community Health Committees, and community dialogue was significantly associated with skilled delivery. These findings were in agreement with a report on strengthening the Community Health Information Systems which established that dialogue days led to increased skilled deliveries.^23^

Moreover, a study investigated the contribution of CHCs on maternal health and the results showed that CHC strengthens and enhances maternal health care and promotes skilled delivery.^24^

### Strength and Weaknesses of the Study

 The first strength of this study is that it is the first study to the best of our knowledge, to assess the association between the functionality of a community unit with skilled birth deliveries and in this study region. The study has linked CU functionality with deliveries to establish that a fully functional community unit provides an opportunity to improve skilled birth deliveries in this region. This calls for a more investment and budgetary allocation to ensure all community units are fully functional. The study further adopted an analytic design and multivariable models to assess the ecological relationship between CHS and Skilled birth deliveries, thereby demonstrating that in resource-poor settings such data can be obtained and analysed to inform policy decisions.

The study has several limitations. Firstly, the influence of CHS functionality on skilled birth delivery is an ecological relationship and is only assessed at 2 levels; the community unit level and at the individual mother's level and the analysis may be affected by ecological fallacy; what happens at the population level is not necessarily what happens to everyone in the population. Secondly, other confounding factors such as education level of mothers, distance to the health facility, cultural and socioeconomic factors which were not included in the analysis because they were not collected. Lastly, the study was purposively done in one-sub-county limiting the level of generalisability.

## CONCLUSION

Community health strategy is an appropriate platform to deliver community-based interventions. Women residing in fully functional health facilities were more likely to deliver in health facilities hence fully functional community units are key to ensure skilled deliveries. Women were more likely to have skilled deliveries if the community units have at least 2 trained CHVs. This is a key emphasis on capacity building for the CHV who influence the health actions of the expectant women. In conclusion, the implementation of a community health strategy is not universal amongst the community health units. Skilled deliveries are still low in the semi-functional community health units. Some of the recommendations of the study would be that there is a need for universal training of the Community Health Committee, Community Health Extension Workers and Community Health Volunteers. There is a need to ensure access to tools by the CHVs particularly the Household register (MOH 513), the service delivery logbook (MOH 514) and referral booklet (MOH 100) for effective service delivery. The government should ensure the improvement of the level of uptake of skilled delivery through cost-effective and sustainable measures to meet the Millennium Development Goal. A future research area could be the cost-effectiveness of the community health strategy on improving skilled birth delivery and the costs of implementation of community health strategies in similar settings.

## DECLARATIONS

### Ethics Approval and Consent to Participate

The proposal was approved by GLUK Research Ethics Committee (GREC) Ref: No. GREC/018/256/2016 on Friday, September 16, 2016. It was also approved by the National Commission for science, technology, and Innovation on 14^th^ June 2017 Ref NACOSTI/p/17/74545/17004. The respondents were interviewed at the household level. Written informed consent was obtained from respondents and each one of them was informed of their rights and obligations. They were informed that there was no risk to risk either legal, social or psychological by participating in the study and an aspect of voluntarism ensured among the participants. The study did not have a medical treatment component. The data collected from study participants werekept strictly confidential and participants were only identified by a code
